# Space-time clustering of childhood leukemia in Colombia: a nationwide study

**DOI:** 10.1186/s12885-020-6531-2

**Published:** 2020-01-20

**Authors:** Laura Andrea Rodriguez-Villamizar, Marcela Pilar Rojas Díaz, Lizbeth Alexandra Acuña Merchán, Feisar Enrique Moreno-Corzo, Paula Ramírez-Barbosa

**Affiliations:** 1 Carrera 32 29-31 Of. 301 Facultad de Salud, Universidad Industrial de Santander, Bucaramanga, Colombia; 2 Instituto Nacional de Salud, Avenida 26 51-120, Bogotá, Colombia; 3 Ministerio de Salud y Protección Social, Cuenta de Alto Costo- Fondo Colombiano de Enfermedades de Alto Costo, Avenida 45 103-34 Of. 802, Bogota, Colombia; 4 Observatorio de Salud Pública de Santander, Fundación Oftalmológica de Santander, Avenida El Bosque 23-60, Floridablanca, Colombia

**Keywords:** Leukemia, Childhood, Cluster analysis, Epidemiology, Colombia

## Abstract

**Background:**

Leukemia is the most common cancer in childhood. The estimated incidence rate of childhood leukemia in Colombia is one of the highest in America and little is known about its spatial distribution.

**Purpose:**

To explore the presence of space-time clustering of childhood leukemia in Colombia.

**Methods:**

We included children less than 15 years of age with confirmed diagnosis of acute leukemia reported to the national surveillance system for cancer between 2009 and 2017. Kulldorff’s spatio-temporal scan statistics were used with municipality and year of diagnosis as units for spatial and temporal analysis.

**Results:**

There were 3846 cases of childhood leukemia between 2009 and 2017 with a specific mean incidence rate of 33 cases per million person-years in children aged 0–14 years. We identified five spatial clusters of childhood leukemia in different regions of the country and specific time clustering during the study period.

**Conclusion:**

Childhood leukemia seems to cluster in space and time in some regions of Colombia suggesting a common etiologic factor or conditions to be studied.

## Background

Leukemia is the most common cancer in childhood. According to the World Health Organization (WHO), the age-standardized cancer incidence rate for 2001–2010 was 140.6 cases per million in children aged 0–14 years, being leukemia the most common cancer (rate = 46.4), followed by tumors of the central nervous system (rate = 28.2), and lymphomas (rate = 15.2) [1]. World estimated childhood leukemia (CL) incidence rates increased 13% in the period 2001–2010 compared to the 1980s rates, and for the recent period, the rates in South America are the highest (33.8 cases per million) in the world, followed by West Asia (33.7 cases per million) [1, 2].

Despite leukemia being the most common cancer in childhood, little is known about its etiology. Genetic, infectious, and environmental factors are the most implicated factors for leukemia. During the last decade, the use of molecular profiling and panel-based testing for detection of germ line syndromes allows the identification of people, and families, with predisposition to hematopoietic malignancies. Furthermore, the WHO included the familial hematopoietic malignancies as an essential component of leukemia diagnosis with reference to specific leukemia predisposition genes [3, 4]. Despite the recognized importance of genetic factors in the occurrence of leukemia, some authors affirm that environmental factors might account for the 85–96% of all cancers in childhood, including leukemia [5–7]. Environmental factors associated with leukemia incidence include ionizing radiation, pesticides exposure, parental smoking, traffic fumes, and household chemicals [8]. The current standardized incidence rates for leukemia in children aged 0–14 years are higher in developing countries where exposure to environmental agents associated with leukemia incidence is probable higher than in developed countries [2]. It is expected that spatial variation in exposure to environmental factors might yield to spatial variations of cancer incidence. Therefore, exploring and describing the spatial distribution and clustering of CL is an important step to explore potential environmental causes related to CL incidence.

Previous studies have described the spatial distribution and clustering of CL in different countries of Europe and North America [9–13]. Most of these studies found evidence of space-time clustering of cases related to the time at birth or the time at diagnosis using different geographical units of analysis (from large administrative areas to exact geocodes or residential locations). These studies have provided evidence that CL tends to cluster in space and time, providing solid bases for further studies focusing on identifying etiologic factors.

The estimated incidence rate of CL in Colombia is one of the highest in Latin America and the Caribbean with an estimated age-standardized rate of 58.4 cases per million of children under 15 years of age during 1992–2013 based on the report of four cancer registries in Colombia [2]. According to the 2017 childhood cancer report of the National Institute for Health in Colombia, there are departments with leukemia incidence rates over 85 cases per million children [14]. National analysis are routinely reporting estimated rates by region and departments but little is known about the spatial distribution of CL in Colombia at a smaller geographic unit; despite the high incidence rates the analysis of potential clustering of CL is not available. Therefore, the objective of this study was to explore the presence of space-time clustering of incident cases of acute childhood leukemia (ACL) in Colombia between 2009 and 2017.

## Methods

### Population and data sources

The study was conducted in Colombia, a country with a population of approximately 48 million of people located at north of South America [15]. The country has 32 departments and 1122 municipalities with administrative and political autonomy. The health system is part of the Social Protection System, which is regulated by the Ministry of Health and Social Protection. The health system was reformed in 1993 conceiving health as a public service to be provided on a market-regulation basis with the participation of health promoting insurance companies (EPS, for its initials in Spanish); during the last decade the main advances of the health system were the expansion of insurance coverage (98% in 2016), the recognition of health as a constitutional right, and the harmonization of health benefit plans for beneficiaries of paid and subsidized insurances systems [16]. Since 2010 the health care for childhood cancer was declared a health priority in Colombia and there is specific regulation for guarantee the timely access to diagnosis, treatment, and rehabilitation [17].

We included incident confirmed cases of ACL (acute lymphoblastic leukemia and acute myeloid leukemia) diagnosed in children less than 15 years old registered in the National Surveillance System of Childhood Cancer (NSSCC) between 2009 and 2017. The NSSCC is a national registry of childhood cancer that started in 2008 as a national surveillance registry for ACL only and in 2014 was converted into the NSSCC, which collects reports for all types of cancer for children and adolescents from 0 to 18 years old in Colombia. The NSSCC is part of the National Surveillance System in Public Health (SIVIGILA, for its name in Spanish) that is led by the National Institute for Health in Colombia [18]. The NSSCC receive reports of probable and confirmed childhood cancers from the health institutions country-wide on a weekly basis. The confirmation of cases is done based on reports of myelogram, immunotypification, histopathology, and cytogenetic tests. The NSSCC compile the reports, eliminate duplicates, and confirm the cases and the date of diagnosis. We obtained anonymous data from the NSSCC that contained the age, sex, the code of the international classification of disease (ICD-10), date of diagnosis, and the municipality of residence at the time of diagnosis for all ACL cases.

In Colombia there are four cancer population-based registries validated by and reporting to the International Agency for Research on Cancer (IARC); these registries are located in the cities of Cali, Bucaramanga, Manizales, and Pasto, and offer high quality information of cancer for these cities; in the case of Bucaramanga the registry includes the four municipalities of the metropolitan area [19]. The cancer registry of Cali was created in 1962, being the oldest cancer registry in Latin America and the pioneer in implementing cancer registry methods in Colombia [20]. The Colombian High Cost Diseases Fund (CAC, for its name in Spanish), a national organization affiliated to the Ministry of Health Protection in Colombia, started in 2015 a National Cancer Registry (NCR), which collects data of all types of cancer cases (children and adults) reported by the health insurance companies, municipalities, and special health regimes, country-wide on a yearly basis [21]. The population coverage in Colombia of the health insurance companies is about 94% [22]; therefore, the NCR was created to be a population-based registry in Colombia. The CAC compile the reports, eliminate duplicates, and confirm the cases and their health service provision by reviewing the medical history and histopathological report for all cancer cases. Thus, the NCR is complementary to other cancer data sources and its main objectives are to assess the cancer risk (magnitude and tendency) and the access to health services. We conducted an analysis of the underreporting of ACL and childhood cancer in the NSSCC, by comparing the ACL reports of the NSSCC with the reports of the cancer population-based registries of Cali and Bucaramanga for both cities during 2010–2015. In addition, we obtained anonymous data from the NCR for 2016 so we were able to conduct a complementary analysis comparing all childhood cancer cases reported to the NCR and the NSSCC during 2016.

Data for the population at risk were obtained from the National Department of Statistics (DANE, for its name in Spanish). Census population projections for children less than 15 years were obtained annually for the study period (2009–2017) for all 1122 municipalities in Colombia [23]. We calculated the geometric centroid coordinates (longitude and latitude) for each polygon of municipality by using the ArcGIS software version 10.3.

### Statistical analysis

Descriptive statistics including mean annual specific incidence rates of ACL and Bayesian smoothed incidence morbidity ratios were calculated by municipality. We used Moran’s Index for calculating global spatial autocorrelation and Kulldorff’s circular scan test to detect local clusters [24]. We used the SaTScan® software version 9.6. In this study we used Kulldorff’s spatio-temporal scan statistics to conduct the first exploratory analysis of childhood cancer clusters in Colombia because: 1) spatial scan statistic are commonly used to detect spatial and/or temporal disease clusters in epidemiological studies and are appropriated for detecting regularly shaped clusters which we expect to find if clusters are related to localized environmental exposures at municipality level; 2) Kulldorff’s scan statistics have very good performance to detect large compact clusters of rare diseases in large territories compared to other scan methods [25]; 3) it has a open software to implement the analysis which make it highly reproducible [26], and 4) it have been widely used for assessing clusters of health events worldwide. We ran the Kulldorff’s test using a retrospective space-time analysis, scanning for clusters with high rates using a discrete Poisson model. The space unit was the municipality of residence and the time unit was the year of diagnosis. The upper limit for the radii was set to include within the circles the 25% of the total number of ACL cases. The significant level for the test was 0.05.

## Results

There were 3915 confirmed incident cases of ACL notified to the SIVIGILA between 2009 and 2017. Nine cases were excluded of the analysis for being residents outside Colombia and 60 cases were excluded because they were identified from a specific department but not at a specific municipality. Therefore 3846 incident cases of ACL were included in the analysis. We identified cases in 629 out of the 1122 municipalities in Colombia (56%).

**Fig. 1:**
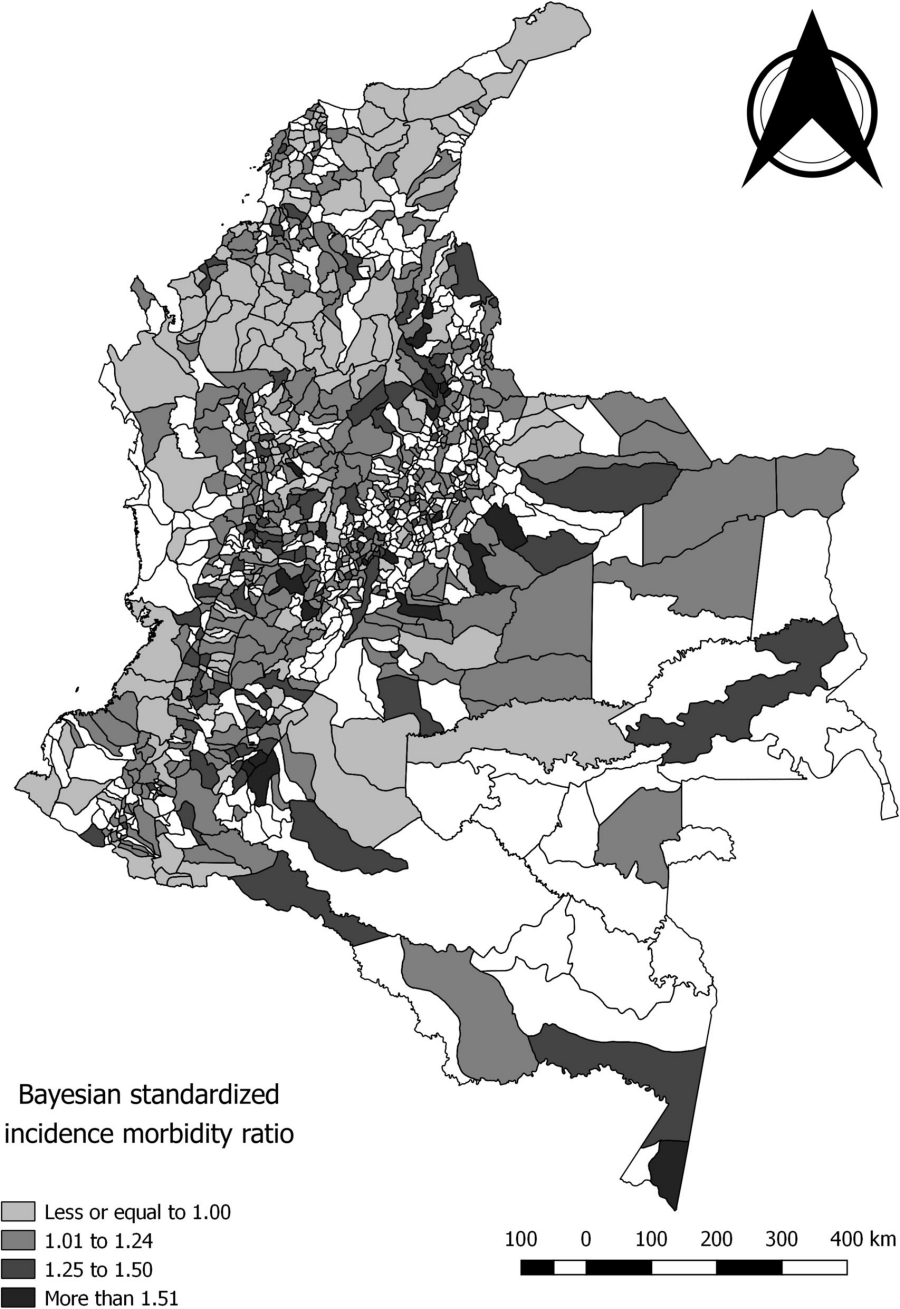
Bayesian smoothed incidence morbidity ratios of acute childhood leukemia by municipality, Colombia, 2009–2017

Most cases were male (54%) and 48% of cases occurred in children under 6 years old. The mean annual incidence rate was 33.15 cases per million for children under 15 years old in Colombia during 2009–2017, and the rate ranged from 24.71 in 2009 to 39.41 in 2016. The district of Bogotá, followed by the departments of Antioquia, Valle, and Santander, were the sites with the highest proportion of cases. The departments of Amazonas, Casanare, and Santander had the highest mean incidence annual rate of ACL during the study period. Bayesian smoothed incidence morbidity ratios by municipality ranged from 0.45 to 2.43 (Fig. 1). Table 1 shows the main characteristics of the study population.

**Table 1:** Characteristics of the study population

Variable	No. cases	% (*n* = 3846)	Cumulative %
Sex			
Female	1763	45.84	45.84
Male	2083	54.16	100
Age (years)			
0	153	3.98	3.98
1	230	5.98	9.96
2	392	10.19	20.15
3	429	11.15	31.31
4	351	9.13	40.43
5	300	7.8	48.23
6	250	6.5	54.73
7	249	6.47	61.21
8	199	5.17	66.38
9	191	4.97	71.35
10	190	4.94	76.29
11	228	5.93	82.22
12	194	5.04	87.26
13	219	5.69	92.95
14	271	7.05	100
	No. cases	%	IR per million
Year of diagnosis			
2009	323	8.4	24.71
2010	355	9.23	27.28
2011	405	10.53	31.24
2012	437	11.36	33.82
2013	452	11.75	35.06
2014	392	10.19	30.45
2015	503	13.08	39.10
2016	507	13.18	39.41
2017	472	12.27	36.66
	No. cases	Population 2013	MIR per million
Department			
Amazonas	17	28,949	65.25
Antioquia	442	1,575,200	31.18
Arauca	23	96,456	26.49
Atlántico	113	650,420	19.30
Bogotá, D.C.	706	1,809,750	43.35
Bolívar	164	616,314	29.57
Boyacá	102	355,591	31.87
Caldas	96	244,793	43.57
Caquetá	51	158,253	35.81
Casanare	63	107,970	64.83
Cauca	108	396,099	30.30
Cesar	74	329,697	24.94
Chocó	18	188,626	10.60
Córdoba	112	520,461	23.91
Cundinamarca	235	713,766	36.58
Guainía	5	15,028	36.97
Guaviare	4	40,556	9.72
Huila	130	339,341	10.96
La Guajira	29	331,338	42.57
Magdalena	36	417,689	9.58
Meta	113	270,616	46.40
Nariño	109	496,285	24.40
Norte de Santander	128	389,382	36.53
Putumayo	24	116,821	22.83
Quindío	39	137,954	31.41
Risaralda	61	231,248	29.31
San Andrés	1	19,197	5.79
Santander	235	512,955	50.90
Sucre	74	252,122	32.61
Tolima	150	392,101	42.51
Valle del Cauca	374	1,093,126	38.02
Vaupés	4	16,683	26.64
Vichada	6	27,502	24.24

*IR* Incidence Rate, *MIR* Mean annual Incidence Rate 2009–2017

### Clustering results

**Fig. 2:**
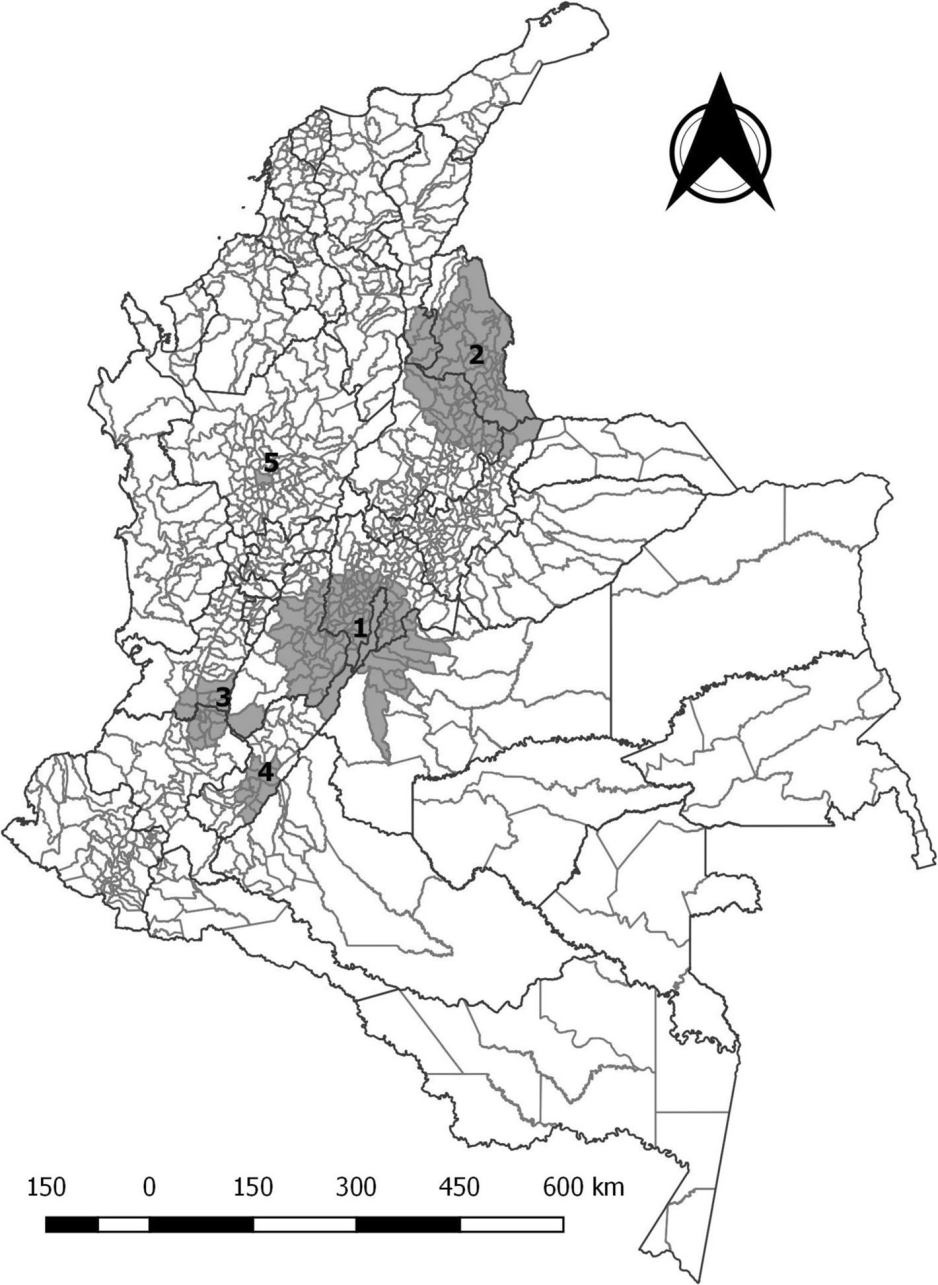
Spatio-temporal clusters of acute childhood leukemia in Colombia, 2009–2017

The Moran’s I was 0.43 (*p* = 0.049) for global spatial autocorrelation of total ACL cases 2009–2017 using municipality as spatial unit. The spatio-temporal scan test identified five clusters with statistical significance. The clusters were of different size and located mainly across the Andean región of the country (Fig. 2). Cluster 1 has the largest number of municipalities and was located at the center of Colombia, including Bogotá, the capital district; this cluster contained 558 ACL cases when a number of 369 cases was expected. Cluster 2 was located at the northeast of the country including a large area of 124 Km and 137 cases in municipalities of Santander and Norte de Santander. Cluster 3 was located at the southwest of the country inlcuding municipalities of Cauca, Valle and Tolima with 135 cases. Cluster 4 was identified in the department of Huila at the south of the country including 30 cases and 11 municipalities. Cluster 5 was identified in the city of Medellín including 62 cases. There were two clusters identified around Leticia in Amazonas, and Cartagena in Bolívar, that did not reach statistical significance in the hypothesis tests. Three out of the five clusters identified ocurred also clustered in time between 2015 and 2017 (Table 2).

**Table 2:** Results of scan test for spatiotemporal clusters of acute childhood leukemia in Colombia, 2009–2017

*Clusters*	*Ratio (Km)*	*N° municipalities*	*RR*	*Time*	*P value*
1	106.9	109	1.60	2013 to 2016	< 0.001
2	124	73	1.95	2015 to 2017	< 0,001
3	48.9	18	1.74	2015 to 2017	0.001
4	43.1	11	3.14	2010 to 2013	0.018
5	0	1	2.10	2015 to 2016	0.021

### Analysis of underreporting for the NSSCC

We compared the number of ACL cases registered in the cancer population-based registries of Cali and Bucaramanga during 2010–2015 with the number of cases of ACL reported to the NSSCC for both cities during the same period. During this period 52 ACL cases were reported for Bucaramanga and 167 for Cali by the cancer registries compared to 51 and 163 reported to the NSSCC, respectively for both cities. Therefore the NSSCC captured and registered 97% of all confirmed incident cases of ACL in both cities during 2010–2015.

A complementary analysis compared the reports of the NCR and the NSSCC for all childhood cancer cases reported during 2016. During this year 1394 incident cases of childhood cancer were identified and 1206 (86.5%) of them were reported to the NSSCC. Among the cases reported to the NSSCC only 54 (3.8%) were discarded due to errors in the notification of diagnosis (not cancer cases) or the year of diagnosis (not incident cases). Therefore the NSSCC captured and registered 83% of all incident cases of childhood cancer in Colombia during 2016.

## Discussion

This nation-wide study assessed the presence of space-time clustering of ACL in Colombia during 2009–2017 using information of the NSSCC. Using Kulldorff’s circular scan test we found five space-time local clusters in different regions of the country during specific time windows. To the best of our knowledge, this is the first study assessing the presence of clusters of ACL at national level in South America.

World childhood leukemia (CL) estimated rates for the period 2001–2010 showed the highest rates in South America (33.8 per million), followed by West Asia (33.7 million) [1]. The estimated incidence rate of CL in Colombia is one of the highest in Latin America and the Caribbean with an estimated standardized rate of 58.4 per million (Colombia four registries 1992–2013) [2]. Using information from the NSCC we found a mean annual incidence rate of 33.15 per million-person year for children under 15 years old for Colombia during 2009–2017. This estimated country rate is similar to the reported by IARC for South America but lower for the estimated rate for Colombia based on the report of the four population-based cancer registries reporting to the IARC. The difference is probably explained by the national coverage of the NSSCC that includes information for all 1122 municipalities in Colombia since 2009, giving a mean estimated for the country and mixing municipalities with high and low incidence of CL. In contrast, the IARC estimates for Colombia are based on four registries that provide high quality information for seven selected municipalities: four in the metropolitan area of Bucaramanga, and the cities of Cali, Pasto and Manizales [19].

The report of ACL cases to the NSSCC is considered of good quality due to the continuous training and quality-assurance process of SIVIGILA in all regions of Colombia. In addition, the complementation of ACL cases report with an active institutional surveillance of ACL-compatible diagnosis favors the identification of ACL cases in early stages [27]. Nevertheless, there is a probability of ACL underdiagnosis that might be mediated by limitations of access to health care facilities in remote rural areas; according to the national study of childhood cancer in 2017 there were no cases of acute lymphoblastic leukemia (82% of total ACL cases) in three departments with remote population (Guainía, Guaviare and San Andres Island). In addition, this study reported that 99.4% of acute lymphoblastic leukemia prevalent cases were covered by health insurance companies, the time between clinical suspicion and the confirmatory diagnosis was on average 21.8 days (median 7 days) and the time between the diagnosis and first treatment was on average 32.1 days (median 3 days) [28].

According to the report of the population-based cancer registry of Cali (the oldest cancer registry in South America) there was an annual increase of 0.7% in the ACL incidence between 1977 and 2011 [29]. The estimated survival proportion for CL is about 55% at 5 years (registries of Colombia 2005–2009) compared to 85% reported by registries in United States during the same period [30]. Taking into account the incidence, trends, and survival of childhood cancer in Colombia, the national goverment have prioritized regulations related to guarantee the health care access, treatment, and follow-up for childhood cancer; however, only few institutions comply with national standards of comprehensive health care for childhood cancer [31]. As part of the national strategies for cancer prevention, treatment and control, the government create the NSSCC starting in 2014 to include the mandatory report of all types of childhood cancer; between 2008 and 2013 the mandatory notification the the national system was exclusive for ACL [18]. Primary prevention of cancer in Colombia is based on tabacco control, HPV vaccination, health style promotion (diet, alcohol, physical activity), and control of exposure to ionizing radiation and known carcinogens in occupational settings (industries) [30]. No specific prevention strategy is directed to environmental exposures related to childhood cancer, probably due to the evidence for those associations still being incipient in the world literature and absent in Colombia.

Childhood leukemia seems to have higher incidence in Latin Americans than in other racial groups. The reported incidence of CL in Guadalajara metropolitan area during 2010–2014 was 64 per million in children under 15 years of age [32]. Similarly, high CL incidence have bee reported in Latin population living in USA [33]. Higher incidence compared to other world regions might be related to genetic factors but also might be related to exposure to environmental factors such as radiation, pesticides, children and parental exposure to toxics, and infections [9, 34–36] that might be present more commonly in Latin American countries compared to developed countries. The occurrence of epidemics of infections disease has been related to space-time clustering of leukemia and other childhood cancers [37, 38]. For leukemia and central nervous system tumors, ionizing radiation in high doses is the only environmental exposure established as a risk factor in the literature [39], while others remained under study such as exposure pesticides, volatile organic compounds such as benzene, and traffic-related air pollution [35, 40–42].

Mapping of disease is part of the descriptive epidemiologic analysis of cancer and any other diseases; however, assessing the pattern of disease occurrence in terms of clustering is less common. Space-time cluster analysis rationale is based on Tobler’s first law of geography, which proposes that “things that are closer to each other are more alike that things that are further apart” [43]. Therefore, assessing space-time clustering helps to uncover disease patterns that might not be evident when routinely mapping the disease using large administrative units for analysis. Furthermore, cluster studies might provide clues of etiologic factors such as common exposures or conditions present in populations. The environmental factors potentially related to ACL show space-time variability and, consequently, variations in exposure to these factors might result in space-time aggregations of ACL cases if the relationship among exposure and ACL is present. Thus, analyzing geographic clusters of ACL might provide key information related to potential etiologic factors.

Most studies assessing clustering of childhood cancer have focused on clustering of leukemia [9, 44]. Childhood leukemia clusters have been identified in different countries but none of these studies have been conducted in South America where the incidence rates of ACL are higher. In United Kingdom, Knox & Gilman studied clustering of CL during 1966 and 1983 and found short-range clustering of ACL at place of notification and at birth and suggested two types of etiologic factors: familiar susceptibility and focal environmental hazards [45]. In Hong Kong, Alexander et al., assessed CL clustering during 1984 and 1990 and found spatial clustering for acute lymphoblastic leukemia suggesting an infectious etiology related to the childhood peak and population mixing [46]. The EUROCLUS project aimed to assess spatial clustering of CL in 17 European countries during 1980–1989 and found evidence of spatial clustering in small census areas [44]. In Switzerland, Kreiss et al., found space-time clustering of childhood leukemia between 1985 and 2010 [11]; they found clustering at birth but not at diagnosis suggesting a common etiologic factor early in life. In Mexico, Tlacuilo-Parra et al., found three spatial clusters of ACL at time of diagnosis within the municipality of Guadalajara during 2010–2014 [32].

Our results found five space-time clusters at time of diagnosis in different regions of the country and three of them were clustered in time after 2015. The spatial clustering might provide information of potential underlying risk factors and the time clustering might provide information related to windows of susceptibility and latent periods [47]. The evidence of spatial clustering at diagnosis for ACL in some regions of Colombia might suggest the presence of potential local environmental exposures which are closer to the time of diagnosis and that the latent periods for those exposures might be relatively short. In this regard, it is important to note that in the four clusters detected that involve different municipalities, the cluster core corresponded to municipalities with predominance of rural areas that are expanded to capital cities such as Bucaramanga, Cali and Bogotá. Most rural municipalities included in these clusters have crops in which the use of pesticides is a common practice [48]. It is suggested that further studies assessing the potential relationship between pesticides’ use and the incidence of ACL might be conducted in those areas. In the case of the city of Medellin, the city itself was identified as a defined spatial cluster with predominant timing between 2015 and 2016. Being that Medellin is a capital city characterized for their manufacturing industry and high levels of intra-urban air pollution, the potential relation of ACL with other potential risk factors such as exposure to volatile organic compounds, other chemicals, or traffic-related air pollution is also matter of further research within the city. A case-control study conducted in the cities of Bogotá and Bucaramanga found an association between parental occupational exposure to carcinogens, especially carcinogenic hydrocarbons before conception, and the occurrence of childhood acute lymphoblastic leukemia [36]. Therefore, occupational exposure to carcinogens and occupational and environmental exposure to pesticides are factors that might explain difference of ACL across regions in Colombia. These factors should be explored in detail by future studies.

In contrast to our findings, other studies did not find any leukemia clustering [49–51]. A recent study conducted by Konstantinoudis et al. [47] assessed spatial clustering of all childhood cancers other than leukemia in Switzerland between 1985 and 2015 and did not found any evidence of space clustering at birth or diagnosis after adjusting for multiple testing of different cancer groups. Lack of clustering of leukemia and other childhood cancers should be interpreted with care. In most cases, divergence of results in epidemiology is seen as lack of consistency and therefore low credibility for associations between potential etiologic factors and diseases [52]. Childhood cancer are a group of diseases that do not have clear etiology and might be related to potential environmental factors that are uneven distributed across places. When assessing spatial distribution of these types of diseases, the heterogeneity of findings must be seen as valuable information about the differential distribution of potential etiologic factors that leads to differential distribution (negative or positive clustering) of disease. Therefore, spatial clustering should be seen as an exploratory analysis that open the window to new potential hypothesis at specific places and should be studied in more detail using other study designs to assess association or causation.

### Strengths and weaknesses

The main strength of this study is that it is a nation-wide study utilizing registries of the NSSCC, which is a national registry with coverage across all municipalities in Colombia. Using the population-base cancer registries as references for underreporting analysis [53], we provided evidence of low percentage (3%) of underreporting of ACL in the NSSCC in two capital cities during 2010–2015; this result implies that the NSSCC is not a fully population based registry but the health care coverage is very high, and therefore the cases included in the NSSCC are a good representation of the total incident cases in Colombia and provide useful information for making geographical analysis and estimations. However, the low percentage of underreporting for ACL observed in the two capital cities does not imply that the same underreporting pattern applies to other non-capital municipalities. We used a long period of analysis (2000–2015) to assess spatial clusters of CL at a reasonable period of time that was not affected by short-term variations of the CL incidence. The use of Kulldorf’s circular spatial scan, the most widely used cluster scan test, let us assess and identify local clusters at municipality level, and the time and quantity of ACL cases provided sufficient power to detect space-time clusters.

We were able to confirm the municipality of residence at time of diagnosis for all cases; however, the residential address and municipality during the pregnancy or at birth was not available from the registries and therefore we were not able to conduct analyses of space-time clustering at an earlier window of susceptibility. The place of residence at diagnosis, however, can be a good representation of earlier place of living, as previous studies on CL in Colombia suggested that residential mobility usually occurs after the time of diagnosis related to access to specialized treatment [54]. Therefore, it is less probable that residential mobility occurs before the diagnosis and, if present, previous studies conducted in Colombia report intra-city mobility more than residential mobility outside the municipality [55]. Spatio-temporal analysis using regional count data may vary depending on the areal and time unit selected (modifiable areal and temporal unit problem) [56]; in our study we used municipality as the unit of analysis which is highly variable in terms of population; therefore the analysis were conducted for rates using as estimates of population at risk the number of population at municipality level. Kulldorff’s scan test assessed only circular shapes and thus clusters of irregular shape might not be adequately identified in this study.

## Conclusions

Acute childhood leukemia seems to cluster in space and time in some regions of Colombia suggesting a common etiologic factor or conditions to be further studied. Future work should be focused on assessing the presence of spatio-temporal clusters of childhood cancer at smaller geographical areas within the regions identified in this study, and assessing the comparison of results using different cluster detection methods. We suggest that adding cluster analysis of disease should be considered as part of the routine surveillance system analysis for childhood leukemia.

## Data Availability

The datasets used and/or analyzed during the current study are available from the corresponding author on reasonable request.

## Abbreviations

ACL: Acute childhood leukemia
CAC: Colombian High Cost Diseases Fund (CAC, for its name in Spanish)
CL: Childhood leukemia
DANE: National Department of Statistics (DANE, for its name in Spanish)
NCR: National Cancer Registry
NSSCC: National Surveillance System of Childhood Cancer
SIVIGILA: National Surveillance System in Public Health (SIVIGILA, for its name in Spanish)
